# Association between nutritional status and anxiety levels in elderly diabetic patients with frailty: a hospital-based cross-sectional study

**DOI:** 10.3389/fnut.2026.1831577

**Published:** 2026-07-08

**Authors:** Danping Peng, Di'en Yan, Yang Guo, Juan Wang, Lingjun Xiao, Qiuxiang Ye

**Affiliations:** 1 Department of Outpatient, Ji'an Central People's Hospital, Ji'an, Jiangxi, China; 2 Department of Endocrinology, Ji'an Central People's Hospital, Ji'an, Jiangxi, China; 3 Department of Rehabilitation Medicine, Ji'an Central People's Hospital, Ji'an, Jiangxi, China; 4 Department of Cardiovascular Medicine, Ji'an Central People's Hospital, Ji'an, Jiangxi, China; 5 Department of Orthopedics, Ji'an Central People's Hospital, Ji'an, Jiangxi, China

**Keywords:** anxiety, diabetes mellitus, elderly, frailty, GAD-7, MNA-SF, nutritional status

## Abstract

**Background:**

Elderly diabetic patients with frailty represent a vulnerable population at heightened risk for both malnutrition and psychological distress. However, the association between nutritional status and anxiety in this specific population remains poorly characterized. This study aimed to investigate the relationship between nutritional status and anxiety levels in elderly diabetic patients with established frailty.

**Methods:**

This cross-sectional study enrolled 318 elderly diabetic patients (age ≥65 years) with confirmed frailty (FRAIL scale ≥3). Nutritional status was assessed using the Mini Nutritional Assessment-Short Form (MNA-SF) and anxiety using the Generalized Anxiety Disorder-7 (GAD-7) scale (score ≥10 indicating clinically significant anxiety). Multivariate logistic regression identified independent associations.

**Results:**

The mean age was 75.20 ± 6.45 years, with 60.4% female. Overall anxiety prevalence was 56.6%, and malnutrition risk (MNA-SF ≤ 11) was present in 83.0% of participants. Anxiety prevalence increased significantly across nutritional categories: 25.9% (normal), 52.1% (at risk), and 80.2% (malnourished; *p* < 0.001). MNA-SF scores were negatively correlated with GAD-7 scores (*ρ* = −0.523, *p* < 0.001). Compared to normal nutritional status, at-risk (aOR = 3.21, 95% CI: 1.57–6.55) and malnourished status (aOR = 12.71, 95% CI: 5.52–29.28) were independently associated with anxiety. Depression and living alone were additional significant predictors.

**Conclusion:**

Poor nutritional status is strongly and independently associated with anxiety in elderly diabetic patients with frailty, demonstrating a clear dose–response relationship. Integrated nutritional and mental health screening is warranted in this vulnerable population.

## 1 Introduction

The global prevalence of type 2 diabetes mellitus (T2DM) among older adults has reached epidemic proportions, with approximately 136 million individuals aged 65 years and older affected worldwide (1). This demographic shift has been accompanied by growing recognition of frailty as a critical geriatric syndrome that substantially impacts diabetes management and outcomes (2). Frailty, characterized by decreased physiological reserve and heightened vulnerability to stressors, affects approximately 30% of elderly diabetic patients and is associated with accelerated functional decline, increased hospitalization, and mortality (3).

Nutritional status represents a fundamental determinant of health in elderly populations, with malnutrition prevalence ranging from 20 to 50% among hospitalized older adults (4). In diabetic patients specifically, the interplay between metabolic dysregulation, altered appetite, and disease-related anorexia creates a particularly challenging nutritional landscape (5). Recent meta-analyses have documented malnutrition prevalence of 33% among diabetic populations, with rates substantially higher in those with concurrent frailty (6). The consequences of malnutrition extend beyond physical deterioration to encompass cognitive impairment, reduced quality of life, and psychological disturbance (7).

Anxiety disorders represent the most prevalent psychiatric conditions among elderly individuals, with estimates suggesting that 15–20% of older adults experience clinically significant anxiety symptoms (8). Among diabetic patients, anxiety prevalence reaches 40–58%, substantially exceeding rates observed in the general population (9). The relationship between diabetes and anxiety appears bidirectional, with anxiety adversely affecting glycemic control through physiological stress responses and behavioral mechanisms, while diabetes-related complications and management burden contribute to psychological distress (10).

Emerging evidence suggests that nutritional status and mental health are intimately connected through multiple pathways, including inflammatory processes, gut-brain axis dysfunction, and micronutrient deficiencies affecting neurotransmitter synthesis (11). Previous studies have demonstrated associations between malnutrition and depression in elderly populations, with odds ratios ranging from 2.0 to 11.0 (12). However, the specific relationship between nutritional status and anxiety—distinct from depression—remains understudied, particularly in the vulnerable subpopulation of elderly diabetic patients with frailty.

Understanding this relationship is clinically imperative for several reasons. First, both malnutrition and anxiety are modifiable conditions amenable to intervention. Second, their co-occurrence may create synergistic negative effects on health outcomes. Third, integrated screening and management approaches could improve efficiency of geriatric care delivery. Critically, no prior study has comprehensively characterized the nutritional-anxiety relationship within this tripartite clinically vulnerable population—elderly patients with concurrent type 2 diabetes and confirmed frailty—particularly within a Chinese tertiary hospital setting. Therefore, this study aimed to investigate the association between nutritional status and anxiety levels in elderly diabetic patients with established frailty, hypothesizing that poorer nutritional status would be independently associated with higher anxiety prevalence.

## 2 Methods

### 2.1 Study design and setting

This cross-sectional study was conducted at the Department of Endocrinology, between January 2023 and December 2024. The hospital serves as a tertiary referral center for a catchment population of approximately 4.8 million residents. The study protocol was approved by the Institutional Review Board of our hospital and written informed consent was obtained from all participants prior to enrollment. The study was conducted in accordance with the Declaration of Helsinki and reported following the Strengthening the Reporting of Observational Studies in Epidemiology (STROBE) guidelines (13). Manuscript preparation and statistical analyses were conducted between January 2025 and February 2026, with submission in March 2026.

### 2.2 Participants

Consecutive patients attending the outpatient diabetes clinic or admitted to the endocrinology ward were screened for eligibility. Inclusion criteria were: (1) age ≥65 years; (2) established diagnosis of type 2 diabetes mellitus according to American Diabetes Association criteria (14); (3) confirmed frailty status defined as FRAIL scale score ≥3; and (4) ability to complete study assessments. Exclusion criteria included: (1) severe cognitive impairment precluding reliable questionnaire completion (Mini-Mental State Examination score <15); (2) acute medical illness requiring intensive care; (3) known diagnosis of psychiatric disorder other than anxiety or depression; (4) current use of antipsychotic medications; and (5) terminal illness with life expectancy <6 months.

Sample size calculation was performed using G*Power 3.1 software (15). Based on previous literature reporting malnutrition-anxiety associations with odds ratios of 2.0–3.0, (12) we estimated a minimum sample size of 280 participants to detect an odds ratio of 2.5 with 80% power at *α* = 0.05, assuming 30% anxiety prevalence and 10 predictor variables in the multivariate model. Accounting for potential incomplete data, we targeted enrollment of 320 participants.

### 2.3 Frailty assessment

Frailty was assessed using the FRAIL scale, a validated five-item screening tool encompassing Fatigue, Resistance (difficulty climbing stairs), Ambulation (difficulty walking), Illnesses (>5 comorbidities), and Loss of weight (>5% in 6 months) (16). Each item is scored 0 or 1, yielding a total score of 0–5. Consistent with established cutoffs, scores of 3–5 were classified as frail, 1–2 as pre-frail, and 0 as robust. Only patients meeting criteria for frailty (score ≥3) were included in the present analysis. The FRAIL scale has demonstrated good diagnostic accuracy in diabetic populations, with area under the curve of 0.71–0.81 (17).

### 2.4 Nutritional assessment

Nutritional status was evaluated using the Mini Nutritional Assessment-Short Form (MNA-SF), a validated screening tool recommended by the European Society for Clinical Nutrition and Metabolism (ESPEN) for geriatric populations (18). The MNA-SF comprises six items assessing food intake decline, weight loss, mobility, psychological stress, neuropsychological problems, and body mass index, yielding a total score of 0–14 points. Participants were categorized according to established cutoffs: normal nutritional status (12–14 points), at risk of malnutrition (8–11 points), and malnourished (0–7 points) (19). The MNA-SF demonstrates sensitivity of 89–100% and specificity of 82–92% for malnutrition detection in elderly populations (20).

Additionally, the Geriatric Nutritional Risk Index (GNRI) was calculated as an objective nutritional indicator using the formula: GNRI = 1.489 × serum albumin (g/L) + 41.7 × (current body weight/ideal body weight) (21). Ideal body weight was calculated using the Lorentz formula. GNRI values <92 indicate major nutritional risk, 92–98 indicate moderate risk, and >98 indicate no risk.

### 2.5 Anxiety assessment

Anxiety was assessed using the Generalized Anxiety Disorder-7 (GAD-7) scale, a brief self-report instrument with established validity in medical populations (22). The GAD-7 comprises seven items rated on a 4-point Likert scale (0 = not at all, 3 = nearly every day), yielding total scores of 0–21. Severity categories include minimal (0–4), mild (5–9), moderate (10–14), and severe (≥15) anxiety (23). Consistent with clinical recommendations, a cutoff score of ≥10 was used to define clinically significant anxiety, which demonstrates sensitivity of 89% and specificity of 82% for generalized anxiety disorder diagnosis (24). The Chinese version of the GAD-7 has been validated in elderly populations with Cronbach’s *α* of 0.92 (25).

### 2.6 Covariates

Demographic variables collected included age, sex, education level (primary, secondary, or higher), marital status, and living arrangement (alone versus with family). Diabetes-related variables included disease duration, current treatment regimen, glycated hemoglobin (HbA1c), and fasting blood glucose (FBG). Comorbidity burden was quantified using the Charlson Comorbidity Index (CCI) (26). Functional status was assessed using the Barthel Index for activities of daily living (ADL) and Lawton scale for instrumental activities of daily living (IADL) (27). Cognitive function was screened using the Mini-Mental State Examination (MMSE), with scores <24 indicating cognitive impairment (28). Depressive symptoms were evaluated using the 15-item Geriatric Depression Scale (GDS-15), with scores ≥5 indicating depression (29). Body mass index (BMI) was calculated from measured height and weight. Polypharmacy was defined as concurrent use of ≥5 medications. Education level was included in Table 1 as a descriptive characteristic. Because education level was not significantly associated with anxiety in univariate screening (*p* = 0.433), it was not entered into the multivariate logistic regression model, consistent with the pre-specified selection criterion of *p* < 0.10.

**Table 1:** Participant characteristics stratified by nutritional status.

Variable	Normal (*n* = 54)	At risk (*n* = 163)	Malnourished (*n* = 101)	*p* value
Age (years)	73.43 ± 6.64	74.79 ± 6.31	76.82 ± 6.28	0.004
Female, n (%)	29 (53.7)	102 (62.6)	61 (60.4)	0.513
Education level, n (%)				0.433
Primary	21 (38.9)	72 (44.2)	47 (46.5)	
Secondary	18 (33.3)	60 (36.8)	39 (38.6)	
Higher	15 (27.8)	31 (19.0)	15 (14.9)	
Living alone, n (%)	15 (27.8)	51 (31.3)	32 (31.7)	0.867
DM duration (years)	11.12 ± 7.68	10.74 ± 7.23	11.59 ± 7.24	0.597
HbA1c (%)	7.88 ± 1.14	7.60 ± 1.24	7.48 ± 1.13	0.120
FBG (mmol/L)	8.76 ± 1.56	8.40 ± 1.48	8.19 ± 1.50	0.079
BMI (kg/m^2^)	26.18 ± 3.74	24.73 ± 3.82	23.87 ± 3.56	<0.001
Albumin (g/L)	37.82 ± 3.79	36.81 ± 3.72	35.53 ± 4.03	0.002
GNRI	97.48 ± 6.15	95.57 ± 5.98	93.40 ± 6.59	<0.001
MNA-SF score	12.91 ± 0.92	9.44 ± 1.07	5.44 ± 1.47	<0.001
GAD-7 score	7.80 ± 3.00	9.56 ± 2.84	12.19 ± 2.61	<0.001
GDS-15 score	4.46 ± 3.10	4.57 ± 2.97	4.74 ± 3.06	0.807
MMSE	25.15 ± 3.35	24.55 ± 3.41	24.30 ± 3.13	0.199
CCI	4.61 ± 1.95	4.61 ± 2.08	4.66 ± 1.95	0.944
Medications, n	6.28 ± 2.39	5.85 ± 2.60	5.87 ± 2.36	0.385
ADL score	70.67 ± 16.38	72.10 ± 17.09	72.09 ± 14.63	0.869
FRAIL score	3.59 ± 0.69	3.74 ± 0.74	3.77 ± 0.77	0.365
Anxiety (GAD-7 ≥ 10), n (%)	14 (25.9)	85 (52.1)	81 (80.2)	<0.001

DM, diabetes mellitus; HbA1c, glycated hemoglobin; FBG, fasting blood glucose; BMI, body mass index; GNRI, Geriatric Nutritional Risk Index; MNA-SF, Mini Nutritional Assessment-Short Form; GAD-7, Generalized Anxiety Disorder-7; GDS-15, 15-item Geriatric Depression Scale; MMSE, Mini-Mental State Examination; CCI, Charlson Comorbidity Index; ADL, activities of daily living.

### 2.7 Statistical analysis

Continuous variables were expressed as mean ± standard deviation or median (interquartile range) as appropriate, and categorical variables as frequencies and percentages. Normality was assessed using the Shapiro–Wilk test. Between-group comparisons utilized the Kruskal-Wallis test for continuous variables and chi-square test for categorical variables.

Correlation between MNA-SF scores and GAD-7 scores was evaluated using Spearman’s rank correlation coefficient. Univariate logistic regression was performed to identify variables associated with anxiety (GAD-7 ≥ 10). Variables with *p* < 0.10 in univariate analysis or those considered clinically relevant *a priori* were entered into multivariate logistic regression models. Results were expressed as odds ratios (OR) with 95% confidence intervals (CI). Model fit was assessed using the Hosmer-Lemeshow goodness-of-fit test. To facilitate direct comparison of effect sizes across variables measured on different scales, continuous variables were standardized by dividing by their respective standard deviations prior to inclusion in the multivariate logistic regression; results for continuous variables in the multivariate model are accordingly reported as adjusted odds ratios per one standard deviation (per SD) change.

Subgroup analyses were conducted stratified by depression status, sex, and age group (young-old: 65–74 years; old-old: ≥75 years) to examine consistency of associations. Sensitivity analyses were performed using alternative anxiety cutoffs (GAD-7 ≥ 8) and GNRI as an alternative nutritional indicator. All statistical analyses were performed using SPSS version 26.0 (IBM Corporation, Armonk, NY) and R version 4.2.0 (R Foundation for Statistical Computing, Vienna, Austria). A two-sided *p*-value < 0.05 was considered statistically significant.

## 3 Results

### 3.1 Participant characteristics

**Figure 1:**
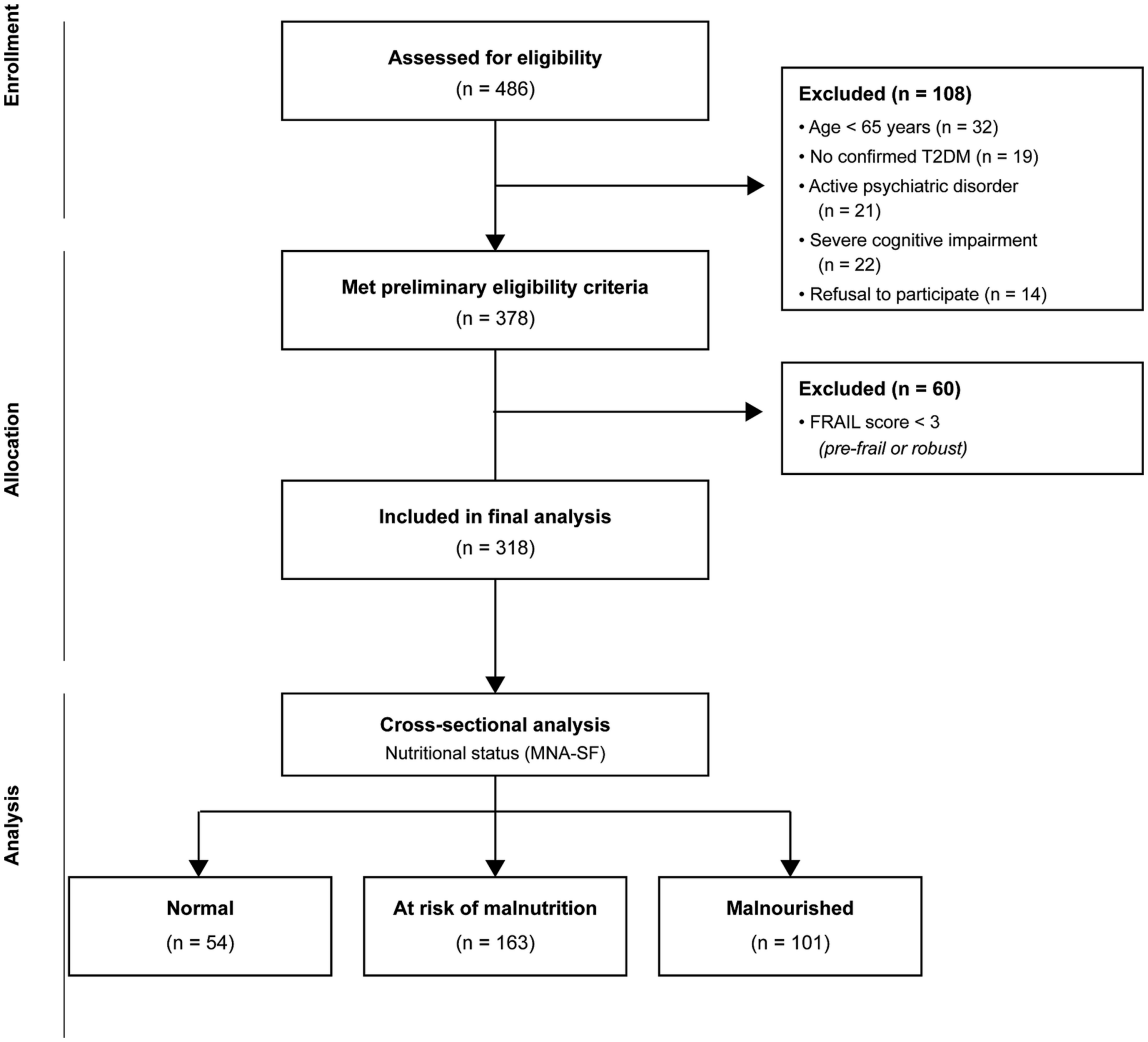
Study flow diagram showing patient screening, eligibility assessment, and final enrollment. Of 486 elderly diabetic patients screened, 318 with confirmed frailty (FRAIL score ≥3) were included in the final analysis.

Of 486 elderly diabetic patients initially screened, 378 met preliminary eligibility criteria. After frailty assessment, 318 patients with confirmed frailty (FRAIL score ≥3) were included in the final analysis (Figure 1). The mean age was 75.20 ± 6.45 years, and 192 participants (60.4%) were female. Mean diabetes duration was 11.08 ± 7.30 years, with mean HbA1c of 7.61 ± 1.19%. The mean FRAIL scale score was 3.73 ± 0.74, confirming moderate-to-severe frailty in the study population.

Regarding nutritional status, 54 participants (17.0%) had normal nutrition (MNA-SF 12–14), 163 (51.3%) were at risk of malnutrition (MNA-SF 8–11), and 101 (31.8%) were malnourished (MNA-SF 0–7). The mean MNA-SF score was 8.75 ± 2.84, and mean GNRI was 95.20 ± 6.34. Overall, 264 participants (83.0%) had some degree of nutritional compromise (MNA-SF ≤ 11).

Concerning anxiety, 180 participants (56.6%) met criteria for clinically significant anxiety (GAD-7 ≥ 10). The mean GAD-7 score was 10.09 ± 3.20. Anxiety severity distribution was: minimal (3.1%), mild (40.3%), moderate (48.7%), and severe (7.9%). Additionally, 147 participants (46.2%) had comorbid depressive symptoms (GDS-15 ≥ 5).

### 3.2 Comparison by nutritional status

Table 1 presents participant characteristics stratified by nutritional status. Patients with poorer nutritional status were significantly older (*p* = 0.004), had lower BMI (*p* < 0.001), lower serum albumin (*p* = 0.002), and lower GNRI (*p* < 0.001). Importantly, GAD-7 scores increased progressively across nutritional categories: 7.80 ± 3.00 (normal), 9.56 ± 2.84 (at risk), and 12.19 ± 2.61 (malnourished; *p* < 0.001).

**Figure 2:**
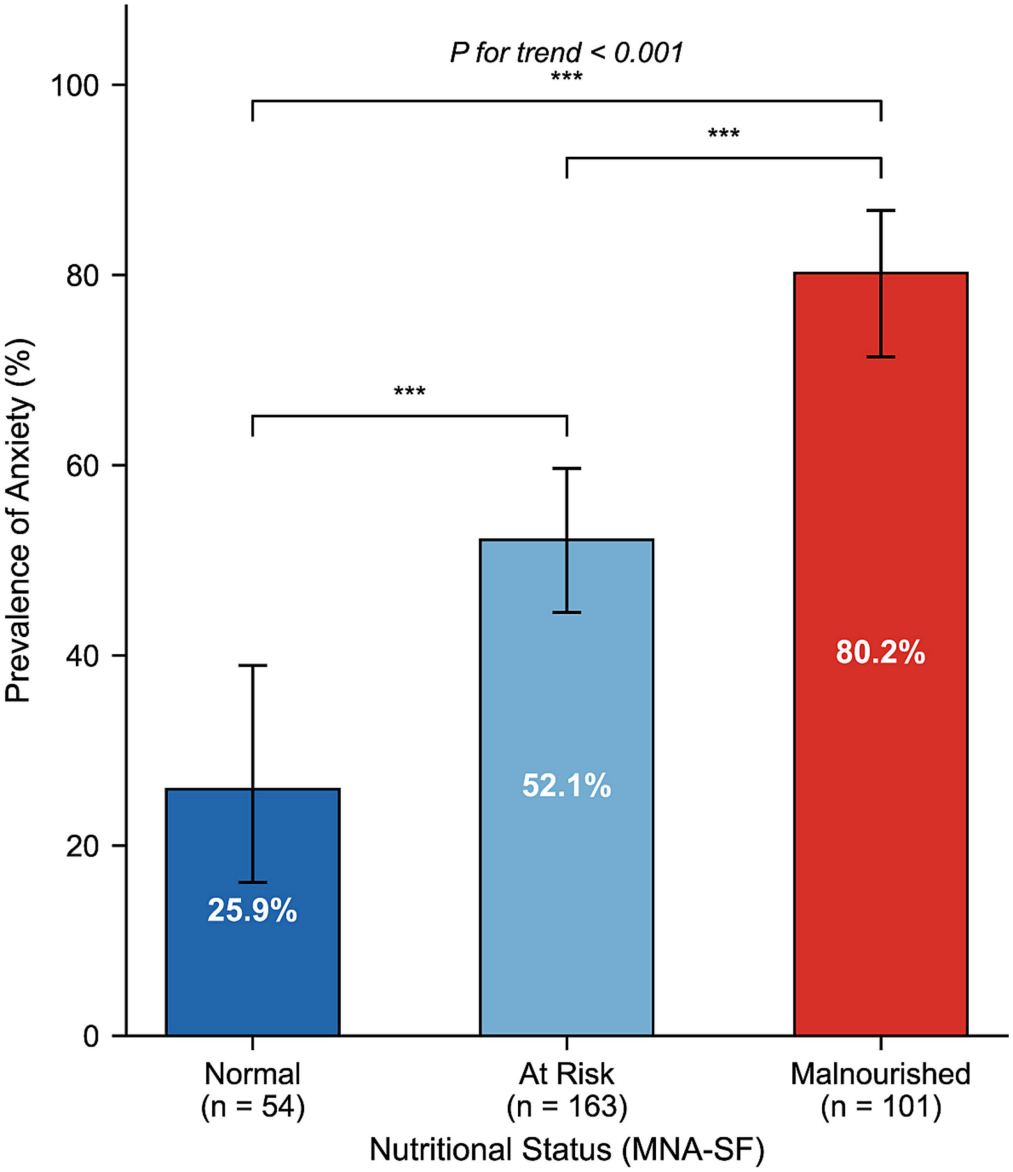
Prevalence of clinically significant anxiety (GAD-7 ≥ 10) by nutritional status category. Anxiety prevalence demonstrated a significant dose–response relationship, increasing from 25.9% in patients with normal nutrition to 52.1% in those at risk and 80.2% in malnourished patients (*P* for trend < 0.001).

The prevalence of clinically significant anxiety (GAD-7 ≥ 10) demonstrated a striking dose–response relationship with nutritional status (Figure 2). Anxiety prevalence was 25.9% among those with normal nutrition, 52.1% among those at risk, and 80.2% among malnourished participants (P for trend < 0.001). Notably, no significant differences were observed across nutritional groups for diabetes duration, HbA1c, depressive symptoms, cognitive function, or comorbidity burden.

### 3.3 Correlation analysis

**Figure 3:**
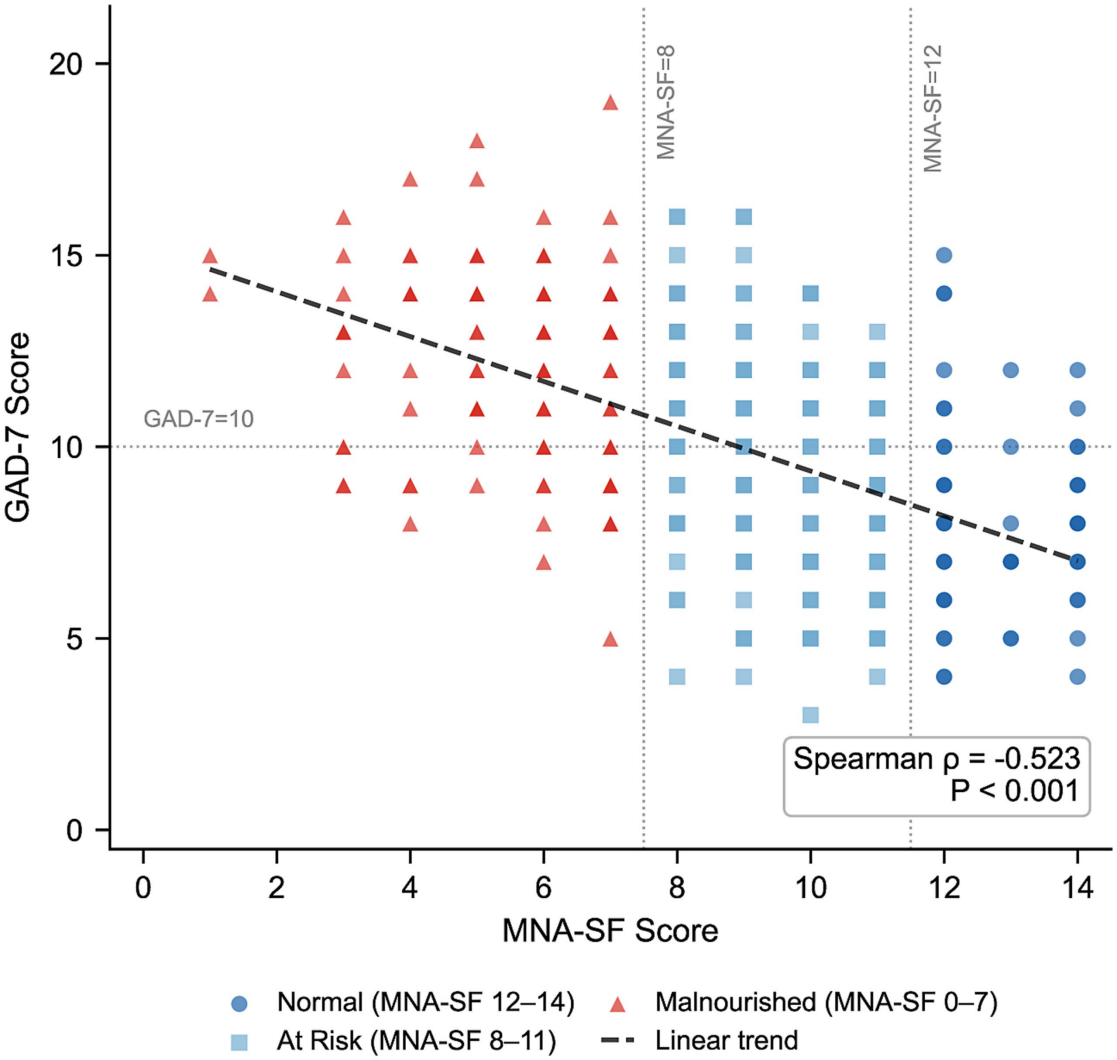
Scatter plot showing the correlation between MNA-SF scores and GAD-7 scores. A significant negative correlation was observed (Spearman *ρ* = −0.523, *p* < 0.001), indicating that better nutritional status was associated with lower anxiety levels. Colors represent nutritional status categories: green (normal), orange (at risk), and red (malnourished).

MNA-SF scores demonstrated a significant negative correlation with GAD-7 scores (Spearman *ρ* = −0.523, *p* < 0.001), indicating that better nutritional status was associated with lower anxiety levels (Figure 3). This correlation was of moderate-to-strong magnitude according to conventional interpretation guidelines. GNRI showed a weaker, non-significant correlation with GAD-7 (ρ = −0.050, *p* = 0.378), suggesting that the MNA-SF may capture nutritional dimensions more relevant to psychological wellbeing than objective biochemical markers alone.

### 3.4 Univariate and multivariate logistic regression

Table 2 summarizes univariate associations with anxiety. Each one-point increase in MNA-SF score was associated with 29% lower odds of anxiety (OR = 0.71, 95% CI: 0.65–0.79, *p* < 0.001). Compared to normal nutritional status, malnourished status conferred dramatically elevated anxiety odds (OR = 11.42, 95% CI: 5.06–25.78, *p* < 0.001). Other significant univariate predictors included living alone (OR = 1.69, *p* = 0.037), lower BMI (OR = 0.93 per kg/m^2^, *p* = 0.014), and depressive symptoms (OR = 2.29, *p* < 0.001).

**Table 2:** Univariate logistic regression analysis for anxiety (GAD-7 ≥ 10).

Variable	OR	95% CI	*p* value
Age (per year)	1.03	0.99–1.06	0.143
Female	1.02	0.65–1.60	0.941
Living alone	1.69	1.03–2.76	0.037
DM duration (per year)	1.01	0.98–1.04	0.515
HbA1c (per %)	0.93	0.78–1.13	0.473
BMI (per kg/m^2^)	0.93	0.87–0.98	0.014
Albumin (per g/L)	0.97	0.92–1.03	0.343
GNRI (per unit)	0.98	0.94–1.01	0.231
MNA-SF (per point)	0.71	0.65–0.79	<0.001
At risk vs. Normal	3.10	1.56–6.16	0.001
Malnourished vs. Normal	11.42	5.06–25.78	<0.001
GDS-15 (per point)	1.21	1.11–1.31	<0.001
Depression (GDS ≥ 5)	2.29	1.45–3.62	<0.001
MMSE (per point)	0.94	0.88–1.01	0.096
Cognitive impairment	1.23	0.78–1.92	0.375
CCI (per point)	1.03	0.92–1.15	0.638
FRAIL score (per point)	1.08	0.80–1.46	0.621

OR, odds ratio; CI, confidence interval; DM, diabetes mellitus; BMI, body mass index; GNRI, Geriatric Nutritional Risk Index; MNA-SF, Mini Nutritional Assessment-Short Form; GDS-15, 15-item Geriatric Depression Scale; MMSE, Mini-Mental State Examination; CCI, Charlson Comorbidity Index.

**Figure 4:**
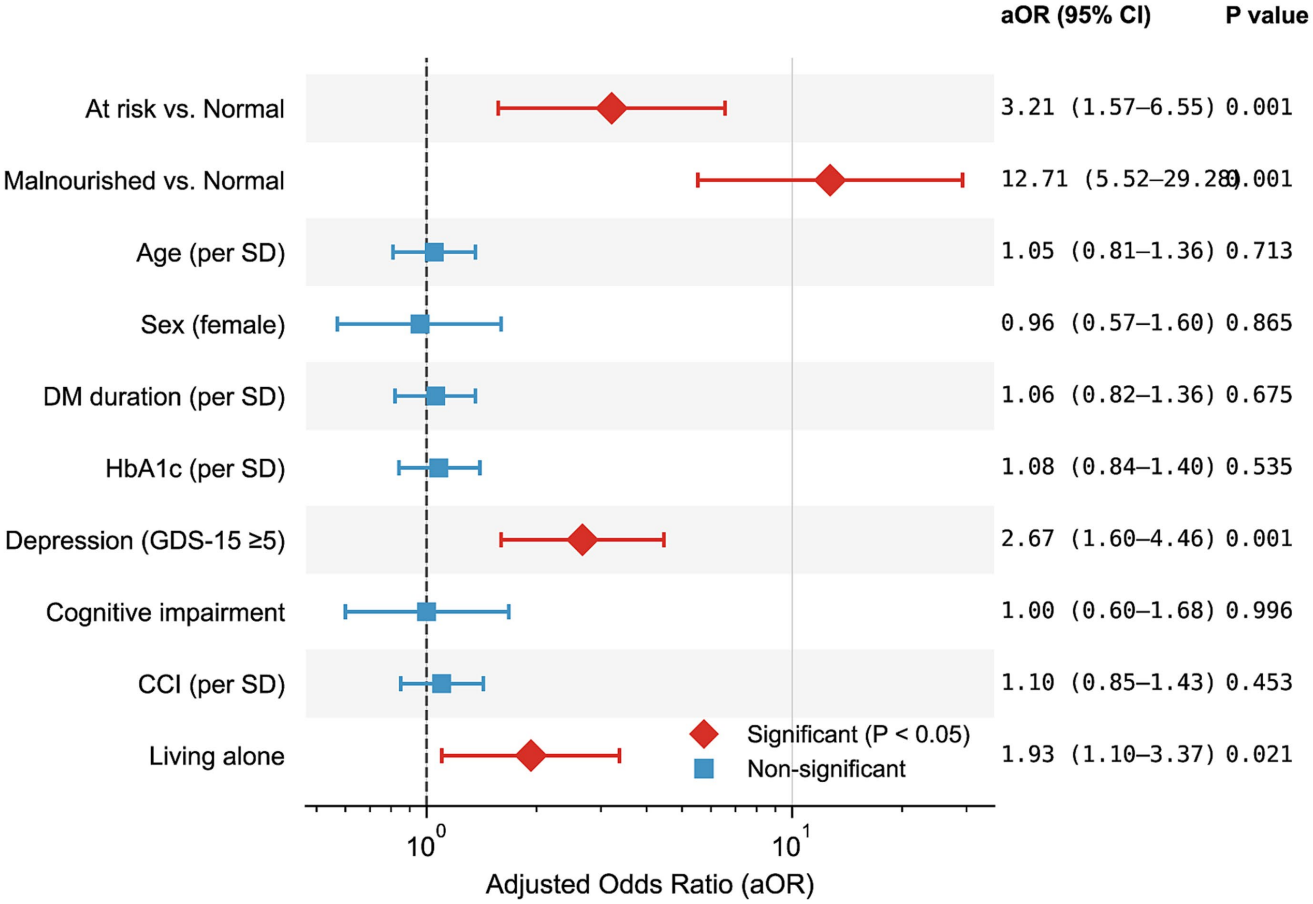
Forest plot of multivariate logistic regression analysis showing adjusted odds ratios (aOR) with 95% confidence intervals for factors associated with anxiety (GAD-7 ≥ 10). Nutritional status categories (at risk and malnourished versus normal), depression, and living alone were significantly associated with anxiety.

In multivariate analysis adjusting for age, sex, diabetes duration, HbA1c, depression, cognitive impairment, comorbidity burden, and living arrangement, nutritional status remained independently associated with anxiety (Table 3; Figure 4). Compared to normal nutritional status, at-risk status was associated with 3.21-fold higher odds of anxiety (aOR = 3.21, 95% CI: 1.57–6.55, *p* = 0.001), while malnourished status was associated with 12.71-fold higher odds (aOR = 12.71, 95% CI: 5.52–29.28, *p* < 0.001).

**Table 3:** Multivariate logistic regression analysis for anxiety (GAD-7 ≥ 10).

Variable	aOR	95% CI	*p* value
At risk vs. Normal	3.21	1.57–6.55	0.001
Malnourished vs. Normal	12.71	5.52–29.28	<0.001
Age (per SD)	1.05	0.81–1.36	0.713
Female	0.96	0.57–1.60	0.865
DM duration (per SD)	1.06	0.82–1.36	0.675
HbA1c (per SD)	1.08	0.84–1.40	0.535
Depression (GDS ≥ 5)	2.67	1.60–4.46	<0.001
Cognitive impairment	1.00	0.60–1.68	0.996
CCI (per SD)	1.10	0.85–1.43	0.453
Living alone	1.93	1.10–3.37	0.021

aOR, adjusted odds ratio; CI, confidence interval; DM, diabetes mellitus; GDS-15, 15-item Geriatric Depression Scale; CCI, Charlson Comorbidity Index; SD, standard deviation. Reference category for nutritional status: Normal (MNA-SF 12–14). Continuous variables in the multivariate model are expressed per one standard deviation (SD) increase following prior standardization.

Additional independent predictors of anxiety included depression (aOR = 2.67, 95% CI: 1.60–4.46, p < 0.001) and living alone (aOR = 1.93, 95% CI: 1.10–3.37, *p* = 0.021). Age, sex, diabetes duration, HbA1c, cognitive impairment, and comorbidity burden were not significantly associated with anxiety after multivariate adjustment. The Hosmer-Lemeshow test indicated adequate model fit (χ^2^ = 8.42, *p* = 0.394). The overall model contained 10 predictor variables and 180 total anxiety events (events-per-variable ratio = 18), satisfying the conventional minimum of ≥10. Nonetheless, because the reference stratum (normal nutritional status) comprised only 14 anxiety events among 54 participants, the absolute effect-size estimate for the malnourished category (aOR = 12.71) should be interpreted with appropriate caution, as sparse data in the reference group can widen confidence intervals and inflate point estimates.

### 3.5 Subgroup analyses

**Figure 5:**
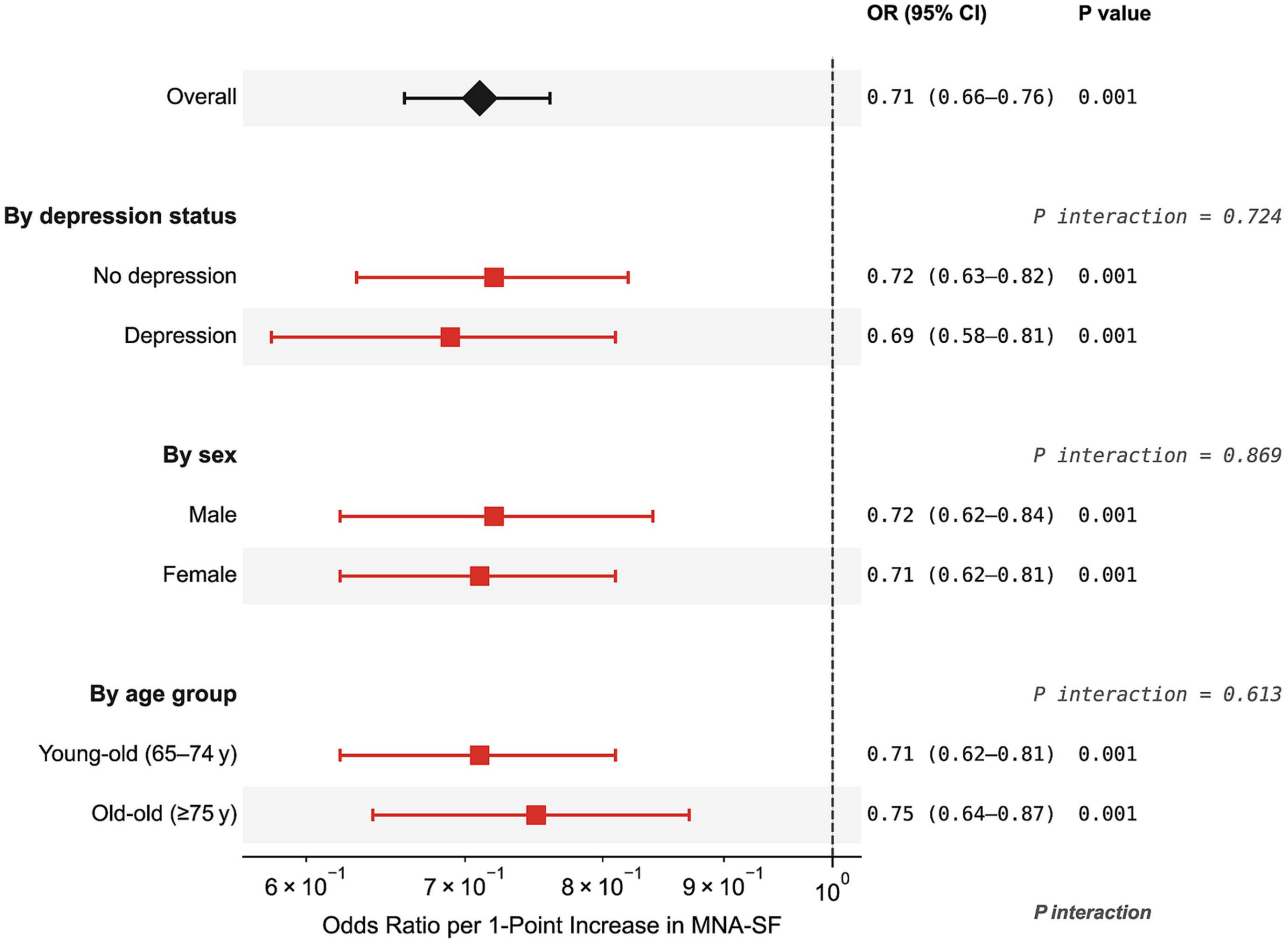
Forest plot of subgroup analyses showing the association between MNA-SF score (per 1-point increase) and anxiety risk. The protective effect of better nutritional status was consistent across subgroups stratified by depression status, sex, and age group.

The protective association between better nutritional status and lower anxiety risk was consistent across all examined subgroups (Figure 5). The association remained significant regardless of depression status (no depression: OR = 0.72, 95% CI: 0.63–0.82; depression: OR = 0.69, 95% CI: 0.58–0.81), sex (male: OR = 0.72, 95% CI: 0.62–0.84; female: OR = 0.71, 95% CI: 0.62–0.81), and age group (young-old: OR = 0.70, 95% CI: 0.60–0.82; old-old: OR = 0.72, 95% CI: 0.63–0.82). No significant interactions were detected between nutritional status and these stratification variables (all P-interaction > 0.05).

## 4 Discussion

This cross-sectional study of 318 elderly diabetic patients with frailty revealed a robust, independent association between nutritional status and anxiety levels. Poor nutritional status, assessed by the MNA-SF, was strongly associated with clinically significant anxiety, with the relationship demonstrating a clear dose–response gradient. Malnourished patients had nearly 13-fold higher odds of anxiety compared to those with normal nutrition, even after comprehensive adjustment for potential confounders. These findings have important implications for clinical practice and highlight the interconnected nature of physical and psychological health in vulnerable geriatric populations.

The high prevalence of both malnutrition risk (83.0%) and anxiety (56.6%) observed in our cohort underscores the substantial burden of these conditions in elderly diabetic patients with frailty. These rates exceed those reported in general elderly diabetic populations, consistent with frailty representing a state of heightened vulnerability across multiple health domains (30). The co-occurrence of nutritional compromise and psychological distress in this population creates a particularly challenging clinical scenario requiring integrated management approaches.

Frailty may play a critical mediating role in the nutrition-anxiety relationship. The frailty syndrome is characterized by accumulated physiological deficits, chronic low-grade systemic inflammation, and dysregulation of the hypothalamic–pituitary–adrenal stress-response axis—all of which can amplify both nutritional vulnerability and psychological distress (30). Beyond biological pathways, frailty intersects with social determinants of health that directly constrain nutritional access: mobility limitations, financial insecurity, loss of cooking independence, and geographical barriers to food procurement are disproportionately prevalent in frail elderly individuals. These structural barriers can perpetuate a vicious cycle in which nutritional deprivation exacerbates anxiety, which in turn suppresses appetite and food-seeking behavior. The independent association between living alone and anxiety observed in the present study aligns with this framework, as social isolation compounds both nutritional risk through reduced meal support and psychological risk through diminished social connection. Future research should examine whether frailty formally mediates the nutrition-anxiety pathway and whether multi-component interventions targeting social barriers to food access attenuate psychological distress in this population.

Our finding of a strong nutrition-anxiety association aligns with and extends previous research examining nutrition-mental health relationships in elderly populations. Hu et al.’s 2024 meta-analysis reported that malnutrition was associated with 2.03-fold increased depression risk in older adults (12). However, anxiety has been less studied than depression, despite representing a distinct clinical entity with potentially different management implications. Our observed effect sizes (aOR 3.21 for at-risk, 12.71 for malnourished) substantially exceed those reported for depression, suggesting that anxiety may be particularly sensitive to nutritional status in this population.

Several mechanistic pathways may explain the nutrition-anxiety relationship. First, micronutrient deficiencies common in malnutrition—including folate, vitamin B12, vitamin D, zinc, and magnesium—directly impact neurotransmitter synthesis and neuronal function (31). These nutrients serve as cofactors for serotonin, dopamine, and gamma-aminobutyric acid (GABA) production, all of which are implicated in anxiety pathophysiology. Second, malnutrition promotes systemic inflammation through elevated pro-inflammatory cytokines (interleukin-6, tumor necrosis factor-*α*, C-reactive protein), which have been linked to anxiety and depression through effects on the hypothalamic–pituitary–adrenal axis and neurotransmitter metabolism (32). Third, gut microbiome alterations associated with poor nutrition may influence brain function through the gut-brain axis, affecting anxiety-related behaviors (33). Furthermore, malnutrition-associated alterations in gut microbiota composition disrupt the bidirectional microbiota-gut-brain axis—a communication network involving neuronal, immune, and endocrine signaling pathways—which has been linked to anxiety and mood regulation (33). Age-related reductions in gastrointestinal absorptive capacity further compound micronutrient deficiency in elderly populations, potentially rendering the oldest-old subgroup disproportionately vulnerable to nutrition-mediated psychological deterioration.

Interestingly, the MNA-SF demonstrated stronger correlation with anxiety than the GNRI, despite the latter’s objective biochemical basis. This observation suggests that the MNA-SF’s multidimensional assessment—incorporating appetite, weight change, mobility, and psychological stress—may capture nutritional dimensions more relevant to mental health than serum albumin-based indices. This finding supports the use of comprehensive nutritional screening tools in geriatric practice rather than relying solely on laboratory parameters.

The identification of depression and living alone as additional independent anxiety predictors is clinically meaningful. Depression and anxiety frequently co-occur, sharing common neurobiological substrates and responding to similar treatments (34). Social isolation, reflected in living alone, is a well-established risk factor for psychological distress in elderly populations, operating through reduced social support, loneliness, and decreased opportunities for health-promoting activities (35). The additive effects of malnutrition, depression, and social isolation suggest that comprehensive geriatric assessment should routinely evaluate all three domains.

The consistency of our findings across subgroups stratified by depression status, sex, and age strengthens confidence in the robustness of the nutrition-anxiety association. The absence of significant interactions suggests that nutritional optimization may benefit anxiety outcomes regardless of these patient characteristics, although individualized approaches remain essential in clinical practice. The absence of a significant interaction with sex indicates that the magnitude of the nutritional-anxiety association did not differ between men and women, suggesting that nutritional optimization represents a broadly applicable strategy for anxiety reduction in this population irrespective of gender. Similarly, although progressive age-related physiological changes—including declining appetite regulation, reduced gastrointestinal absorptive capacity, and accumulating comorbidities—may incrementally amplify nutritional vulnerability, the association between nutritional status and anxiety did not differ significantly between the young-old (65–74 years) and old-old (≥75 years) subgroups, a finding that warrants further longitudinal investigation.

Our study has several strengths. First, we focused specifically on elderly diabetic patients with confirmed frailty, a well-defined vulnerable population with high clinical relevance. Second, we employed validated, internationally recognized instruments for both nutritional (MNA-SF) and anxiety (GAD-7) assessment. Third, comprehensive adjustment for potential confounders, including depression, cognitive function, and comorbidity burden, strengthens causal inference. Fourth, subgroup analyses demonstrated consistent findings across clinically relevant strata.

Several limitations warrant acknowledgment. First, the cross-sectional design precludes causal inference; longitudinal studies are needed to establish temporality of the nutrition-anxiety relationship. Second, our single-center design may limit generalizability to other settings or populations. Third, anxiety was assessed by self-report questionnaire rather than structured psychiatric interview, potentially leading to misclassification. Fourth, we could not assess certain potentially relevant factors such as dietary quality, physical activity patterns, or social support networks. Fifth, the observational nature of our study cannot exclude residual confounding by unmeasured variables. Sixth, although education level was collected and is reported in Table 1, its non-significant univariate association with anxiety (*p* = 0.433) precluded its inclusion in the multivariate model; nonetheless, residual educational confounding cannot be fully excluded. Seventh, the reference stratum of normal nutritional status comprised only 14 anxiety events; while the overall events-per-variable ratio (EPV = 18) satisfied the conventional minimum, sparse-data bias in this stratum may have contributed to the wide confidence interval and potentially inflated point estimate for the malnourished category, and absolute effect-size estimates should therefore be interpreted with caution.

From a clinical perspective, our findings support routine screening for both malnutrition and anxiety in elderly diabetic patients with frailty. Given the strong association observed, nutritional interventions may have beneficial effects on psychological wellbeing in addition to physical health outcomes. Conversely, addressing anxiety may improve appetite and nutritional behaviors. Future research should examine whether nutritional optimization reduces anxiety in prospective trials and whether combined nutritional-psychological interventions offer synergistic benefits in this population. Specifically, integrated geriatric care protocols should incorporate routine MNA-SF screening at every clinical encounter, with standardized referral thresholds triggering dietitian consultation when MNA-SF scores fall to ≤11. Concurrent GAD-7 anxiety screening is warranted given the strong bidirectional relationship observed. Multi-component nutritional interventions—including individualized dietary counseling, oral nutritional supplementation where indicated, and practical support such as community meal delivery programs and caregiver training in meal preparation—should be positioned as integral components of comprehensive psychological care, not merely physical health measures. Addressing modifiable social barriers to adequate nutrition, including food insecurity, mobility limitations, and social isolation, may yield concurrent benefits for both nutritional status and psychological wellbeing. Caregiver education programs that build capacity for early recognition of malnutrition signs and anxiety symptoms could further facilitate timely intervention in home and residential care settings.

## 5 Conclusion

In this cross-sectional study of elderly diabetic patients with frailty, poor nutritional status was strongly and independently associated with anxiety, demonstrating a clear dose–response relationship. Malnourished patients had approximately 13-fold higher odds of clinically significant anxiety compared to those with normal nutrition. These findings underscore the importance of integrated nutritional and mental health screening in geriatric diabetes care. Prospective studies are needed to determine whether nutritional interventions can effectively reduce anxiety burden in this vulnerable population.

## Data Availability

The original contributions presented in the study are included in the article/supplementary material, further inquiries can be directed to the corresponding author.
